# Alumina Coated Silica Nanosprings (NS) Support Based Cobalt Catalysts for Liquid Hydrocarbon Fuel Production From Syngas

**DOI:** 10.3390/ma12111810

**Published:** 2019-06-04

**Authors:** Abdulbaset Alayat, Elena Echeverria, Farid Sotoudehniakarani, David N. Mcllroy, Armando G. McDonald

**Affiliations:** 1 Renewable Materials Program, Department of Forest, Rangeland and Fire Sciences, University of Idaho, Moscow, ID 83844-1133, USA; alay0843@vandals.uidaho.edu (A.A.); farids@uidaho.edu (F.S.); 2 Department of Physics, Oklahoma State University, Stillwater, OK 74078-3072, USA; elena.echeverria@okstate.edu (E.E.); dave.mcilroy@okstate.edu (D.N.M.)

**Keywords:** alumina, cobalt catalyst, Fischer–Tropsch synthesis, hydrocarbons, silica nanosprings

## Abstract

The effects of Al_2_O_3_ coating on the performance of silica nanospring (NS) supported Co catalysts for Fischer–Tropsch synthesis (FTS) were evaluated in a quartz fixed-bed microreactor. The Co/NS-Al_2_O_3_ catalysts were synthesized by coating the Co/NS and NS with Al_2_O_3_ by an alkoxide-based sol-gel method (NS-Al-A and NS-Al-B, respectively) and then by decorating them with Co. Co deposition was via an impregnation method. Catalysts were characterized before the FTS reaction by the Brunauer–Emmett–Teller (BET) method, X-ray diffraction, transmission electron microscopy, temperature programmed reduction, X-ray photoelectron spectroscopy, differential thermal analysis and thermogravimetric analysis in order to find correlations between physico-chemical properties of catalysts and catalytic performance. The products of the FTS were trapped and analyzed by GC-TCD and GC-MS to determine the CO conversion and reaction selectivity. The Al_2_O_3_ coated NS catalyst had a significant affect in FTS activity and selectivity in both Co/NS-Al_2_O_3_ catalysts. A high CO conversion (82.4%) and Σ > C_6_ (86.3%) yield were obtained on the Co/NS-Al-B catalyst, whereas the CO conversion was 62.8% and Σ > C_6_ was 58.5% on the Co/NS-Al-A catalyst under the same FTS experimental condition. The Co/NS-Al-A catalyst yielded the aromatic selectivity of 10.2% and oxygenated compounds.

## 1. Introduction

Fischer–Tropsch Synthesis (FTS) has been recognized as a promising route to produce environmentally clean liquid fuels and industrial chemicals (heavy and light hydrocarbons) from renewable feed-stocks [1]. FTS is a heterogeneous catalyzed polymerization reaction of syngas (mixture of CO and H_2_), which is derived from the gasification of a variety of feed-stocks (natural gas, coal, and biomass), into a wide range of molecular weight hydrocarbon chains. The stoichiometry of FTS reactions can be written as Equations (1 and 2):

nCO + (2n +1)H_2_ → C_n_H_2n+2_ + nH_2_O (1)

nCO + 2nH_2_ → C_n_H_2n_ + nH_2_O (2)

For this reaction, several Group VIII transition metals, such as iron (Fe), cobalt (Co) and ruthenium (Ru), are all active in FTS, but only Fe and Co are used for industrial application because of their high activity, low methane selectivity, low cost and high water gas shift (WGS) activity [1,2,3]. The FTS efficiency and hydrocarbon product distribution depend on variables, such as catalyst specifications (e.g., nature and composition of the catalyst, promoters, support, etc.) and processes conditions (e.g., reactor type, temperature and reaction pressure, activation and preparation methods, etc.) [1,4]. The type of inorganic support (e.g., SiO_2_, Al_2_O_3_, TiO_2_, activated carbon, zeolite, etc.) plays a key role in the control of catalytic activity, reducibility and the product distribution for FTS [5,6]. It is known that Co has a low dispersion on SiO_2_ support because of the weak interaction between Co nanoparticles and silica, which leads to agglomeration of Co particles. This problem can be overcome by modifying or coating the silica support with an oxide promoter such as Al_2_O_3_. The use of these metal oxides supports can improve the surface properties such as dispersion of Co on the support [3,6,7].

Coating the silica support can enhance the catalytic activity of the Co catalysts by changing properties like the surface acidity [5,8]. A number of studies have shown improved activity and/or selectivity of Co-based FTS catalysts with silica supports by mixing with Al_2_O_3_ [6,7]. For example, Zhang et al. [6] and Savost’yanov et al. [5] studied the effect of Al_2_O_3_ promoter on the FTS performances of the Co/SiO_2_ catalyst. They found that the addition of Al_2_O_3_ onto the Co/SiO_2_ catalyst significantly improved the activity and selectivity to >C_5_ hydrocarbons by adjusting surface properties such as Co dispersion and reduction temperature. To date, we are unaware of FTS studies using Al_2_O_3_ to modify 1 dimensional (1D) nanostructured supports, such as silica nanosprings (NS). Silica NS are a new 1D support materials for catalysts, and have been demonstrated to meet the criteria of supports for FTS applications [1,2,9].

Therefore, the objective of the present study is to investigate the effects of the Al_2_O_3_ coating on the physico-chemical properties and the catalytic performance of Co/NS catalysts during FTS. The properties of prepared catalysts were comparatively characterized by various analytical techniques such as surface area, hydrogen temperature programmed reduction (H_2_-TPR), X-ray diffraction (XRD), transmission electron microscopy (TEM), X-ray photoelectron spectroscopy (XPS), differential thermal analysis (DTA), Fourier transform infrared spectroscopy (FTIR) and thermogravimetric analysis (TGA). In addition, the CO conversion and hydrocarbon selectivity of the FTS catalysts were determined by gas chromatography (GC) and GC-mass spectrometry (GC-MS) analyses.

## 2. Experimental Methods

### 2.1. Preparation of Catalysts

The silica NS were synthesized in 0.5 g batches by using a gold-catalyzed vapor–liquid–solid growth technique, then heated at 600 °C for 5 h in order to remove any residual precursors on the support according to previous reports [2] and Wang et al. [1]. The NS were dried over night at 110 °C before use. The 15 wt% Co/NS catalyst was prepared by the conventional incipient wetness impregnation (IWI) method. To the prepared 15 wt% Co/NS catalyst, the dried NS support (79.5 mg in 15 mL of ethanol) was impregnated at 70 °C to incipient wetness with an aqueous solution containing Co(NO_3_)_2_·6H_2_O (70 mg in 15 mL of water).

The Al_2_O_3_ nanoparticle support (control) was prepared by an alkoxide based sol-gel method [10]. Aluminum tri-sec-butoxide (Al (O-s-Bu)_3_) as an aluminum precursor (12 mmol) was dissolved in isopropanol (C_3_H_8_O, 150 mmol) with acetylacetone (C_5_H_8_O_2_, 10 mmol) at 20 °C, and then ultra-sonicated for 1 h at 50 °C. Nitric acid (0.4 mL) and ethanol (100 mmol) were added drop-wise to the aluminum precursor solution (0.5 mL). After cooling, a viscous gel was obtained. The Al_2_O_3_ gel solution was vigorously stirred for 30 min, and then aged for 8 d at room temperature. For the synthesis of 15 wt% Co/Al_2_O_3_ catalyst, the aqueous solution containing Co(NO_3_)_2_·6H_2_O (75 mg in 15 mL of water) was added dropwise under stirring at 70 °C onto the solution of Al_2_O_3_ support precursor (85.3 mg).

**Figure 1:**
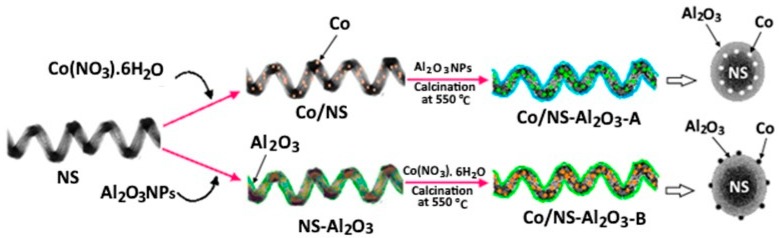
Schematic showing the preparation of the two Co/NS-Al_2_O_3_ catalysts: Co/NS-Al-A and Co/NS-Al-B.

For the synthesis of 15 wt% Co/NS-Al_2_O_3_ catalyst, two types of catalysts were prepared in this study (Figure 1). The process for preparing the first type (A) of Co/NS-Al_2_O_3_ catalyst was by impregnating an aqueous solution of Co(NO_3_)_2_·6H_2_O (88 mg in 15 mL of water) onto the NS (100 mg in 15 mL of ethanol) support at room temperature. Then, the solution of Al_2_O_3_ gel (47 mg) was added dropwise to the Co/NS and labeled Co/NS-Al-A. The second type (B) Co/NS-Al_2_O_3_ catalyst was made by adding a solution of Al_2_O_3_ gel (47 mg) onto the NS (100 mg in 15 mL of ethanol) support at room temperature, as described by the chemical Equation (3) as follows:

3Al_2_O_3_ + 2SiO_2_ (NS) → 3Al_2_O_3_.2SiO_2_ (NS) (3)

Then, Co(NO_3_)_2_·6H_2_O (88 mg in 15 mL of water) solution was added to the NS-Al_2_O_3_ suspension, and the catalyst was named Co/NS-Al-B. All prepared (Co/NS-Al-A, Co/NS-Al-B and Co/Al_2_O_3_) catalyst suspensions were stirred at 70 °C for 12 h, dried at 110 °C overnight and then the obtained catalysts were calcined immediately in the air at 550 °C for 5 h.

### 2.2. Characterization of As-Prepared Catalysts

The reducibility of the calcined catalysts was studied by H_2_-TPR using a ChemiSorb 2720 chemisorption analyzer (Micromeritics, Norcross, GA, USA) equipped with a thermal conductivity detector (TCD). For TCD calibration, CuO (20 mg, 99.99%) was reduced between 25 and 500 °C. Prior to the H_2_-TPR measurement, the catalyst was pretreated in a N_2_ flow of 30 mL min^−1^ at 150 °C for 1 h to remove surface impurities, and then cooled down to room temperature. Subsequently, 5 vol.% H_2_ in N_2_ atmosphere (30 mL min^−1^) was passed through the catalyst then ramped from room temperature to 1000 °C at a heating rate of 10 °C min^−1^. The Brunauer−Emmett−Teller surface area (S_BET_) measurements of all degassed (220 °C for 30 min) catalysts (60 mg) were determined by an N_2_ adsorption-desorption isotherm at −196 °C using a FlowSorb II 2300 instrument (Micromeritics, Norcross, GA, USA).

FTIR measurement of the calcined catalysts (10% in KBr powder) was recorded in the 500–3500 cm^−1^ range using a diffuse reflectance (5% in KBr) accessory on an iS10 spectrometer (ThermoNicolet, Madison, WI, USA). The TGA and DTA analysis of the calcined catalysts (5 mg) were performed, respectively, on a Perkin Elmer TGA-7 instrument and a DTA-7 instrument from room temperature to 900 °C at a rate of 20 °C min^−1^ in a flow of N_2_ (30 mL min^−1^).

The XRD pattern of the calcined catalysts was obtained with a Siemens Diffractometer D5000 using monochromatic Cu/kα radiation at an X-ray wavelength (λ) of 0.1540 nm. The diffraction intensities were collected within 2θ range of 10–80° with 0.01° step and a 1 s acquisition time per step. To calculate the average crystallite size of Co_3_O_4_, the Debye–Scherrer’s equation was employed as [11]:

(4) $$\;\;\;d_{XRD}=\frac{k\lambda }{\beta_{hkl}\cos\theta }$$

where *d*_XRD_ is the average crystallite in nm; K is a constant related to crystallite shape, normally taken as 0.9; λ is the X-ray wavelength (λ = 1.54 Å); β is line broadening at half the maximum intensity (FWHM) in radians and θ is the angular position of the peak of interest. The average particle size of *d*(Co^0^) was calculated from the *d*_XRD_(Co_3_O_4_) according to following formula [12]:

(5) $$d_{XRD}(Co^{0})=0.75\;d_{XRD}\left(Co_{3}O_{4}\right)$$

where *d*_XRD_(Co^0^) is an average particle size in nm of Co metal and *d*_XRD_(Co_3_O_4_) is the average particle size of Co oxide. The Co^0^ metal dispersion (*D*_XRD_) was estimated by assuming a spherical geometry of the metal particles with a uniform site density of 14.6 atoms/nm^2^ using the following formula [13]:

D = [96/d_XRD_ (Co^0^)] (6)

where D is the % Co^0^ dispersion and *d*_XRD_ (Co^0^) is the mean particle size of Co^0^ in (nm).

The microstructures and morphology of prepared catalysts (dispersed in ethanol and applied to a copper grid coated with carbon support film) was characterized by TEM (JEOL JEM-2100 or JEM-2010, JEOL USA Inc., Peabody, MA, USA), operated at 200 kV. The cobalt particle size (d_TEM_) from TEM micrographs was measured using ImageJ software (version 1.52).

The XPS scans of powder samples were acquired with a custom built ultrahigh vacuum (UHV) chamber using the monochromatic Al K-α radiation (1486.6 eV) of a dual anode X-ray source, XR 04-548 from PHYSICAL ELECTRONICS, and the kinetic energy of the photoelectrons was measured with an EA 125 hemispherical electron energy analyzer (Scienta Omicron, Taunusstein, Germany) with a resolution of 25 meV.

### 2.3. Catalytic Activation and FTS Evaluation

FTS experiments were of the calcined Co/NS, Co/Al_2_O_3_, Co/NS-Al-A and Co/NS-Al-B catalysts were carried out in a quartz fix-bed micro-reactor (10 mm Ø × 300 mm with a “0” quartz frit connected 180 mm from the top to support the catalyst) housed in a tube furnace (25 mm Ø × 150 mm). In each FTS run, 20 mg of calcined catalyst was placed in the reactor and mixed with 40 mg quartz sand. The catalysts were activated in a flow of (H_2_/N_2_ = 40/60 mL min^−1^) gas mixture metered with mass flow controllers (CG1, Dakota Instruments). The reactor temperature was increased from 25 to 700 °C and held there for 24 h. The activated catalyst was cooled to 150 °C and subsequently used for in-situ FTS reaction. After catalyst activation, the gases (H_2_/CO/ N_2_ = 60/30/10 mL min^−1^) were metered with mass flow controllers (CG1, Dakota Instruments) at atmospheric pressure and fed to the reactor at 230 °C. The liquid products were collected in a three-stage impinger trap placed in a liquid nitrogen bath. During the reaction, the tail gas was collected in a Tedlar^TM^ PVF (300 × 300 mm^2^) gas-sampling bag. FTS reaction was operated for 34 h under reaction temperature of 230 °C. The outlet gas composition was analyzed by GC-TCD (Series 350, GOW-MAC Instrument Co., Bethlehem, PA, USA) with (i) a packed HaySep DB stainless steel column (3.3 mm Ø × 9.1 m) at 25 °C for CO, CO_2_, H_2_, N_2_ and CH_4_, and (ii) a packed PoraPakQ stainless steel column (3.3 mm Ø × 1.8 m) at 60 °C for C_x_H_y_ (x ≤ C4) with He elution (30 mL min^−1^). The liquid products C_x_H_y_ (x ≥ C5) collected were then identified by GC-MS (Focus-ISQ, ThermoElectron, West Palm Beach, FL, USA). Separation was achieved with a ZB5ms (0.25 mm Ø × 30 m, Phenomenex) capillary column with a temperature program of 40 °C (1 min) ramped to 250 °C at 5 °C min^−1^. Data was analyzed using the Xcalibur v4 software. The identity of the compounds was determined with n-alkane standards (C_6_ to C_30_) and mass spectral matching with the NIST 2017 mass spectral library. H_2_, CO conversions (%) and product selectivities (%) were calculated based on previous studies [1,4,14_ENREF_19].

## 3. Results and Discussion

### 3.1. Catalyst Preparation and Characterization

Four different catalysts (Co-NS, Co/Al_2_O_3_, Co-NS-Al-A and Co-NS-Al-B catalysts) were prepared in order to examine the effect of the addition of alumina to the NS supports for FTS. TEM micrographs of prepared silica NS are shown in Supplementary Figure S1. The S_BET_ of calcined catalysts were determined and given in Table 1. The S_BET_ for the Co/NS and Co/Al_2_O_3_ catalysts were 193 and 108 m^2^ g^−1^, respectively, which were lower than their supports of 314 and 227 m^2^ g^−1^, respectively, while the calcined Co/NS-Al-A and Co/NS-Al-B catalysts were higher at 199 and 260 m^2^ g^−1^, respectively. The addition of Al_2_O_3_ to the Co/NS catalyst did not noticeably change the surface area of the Co/NS-Al-A catalyst, whereas, the surface area was higher by applying Al_2_O_3_ to the NS support in the Co/NS-Al-B catalyst. This seems to indicate that the addition of Al_2_O_3_ species to the Co/NS-Al-B catalyst improved the dispersion of Co oxides particles on the surface, as well as the porous structure (space limitation inside the pores) of these oxides and, thus, increased the SBET value of the Co/NS-Al-B catalyst [4,7]. Furthermore, the Co/NS-Al-B catalyst shows slightly higher surface dispersion (16%) than other catalysts due to the strong Co−NS interaction with modification of Al_2_O_3_ species, which lead to the small particle size of Co_3_O_4_ species and higher supported Co dispersion. Very similar results were obtained using the Co/SiO_2_ catalysts promoted with an Al_2_O_3_ support [6]. This result is probably attributable to the fact that the interactions of Co oxides with Al_2_O_3_ are stronger that those between Co oxides and SiO_2_ NS [6].

**Figure 2:**
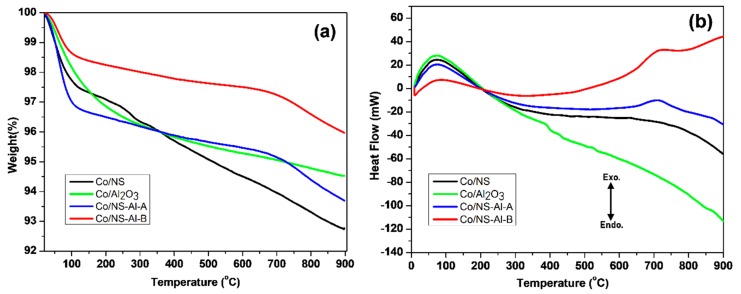
(**a**) Thermogravimetric analysis (TGA) and (**b**) DTA thermograms of the calcined Co/NS, Co/Al_2_O_3_, Co/NS-Al-A and Co/NS-Al-B catalysts.

Thermogravimetric analysis (TGA) was carried out to determine the thermal stability of the calcined Co/NS, Co/Al_2_O_3_, Co/NS-Al-A and Co/NS-Al-B catalysts. The TGA thermograms of the calcined Co/NS, Co/Al_2_O_3_, Co/NS-Al-A and Co/NS-Al-B catalysts (Figure 2a) indicates that the overall weight loss up to 900 °C were 6.9%, 6.3%, 5.4% and 4.0%, respectively. The first stage mass loss (about 3%) of all catalysts occurred between the temperature range of 30–150 °C, which is likely due to evaporation of adsorbed water, solvent and organic compounds on the catalysts; this is supported by an exothermic peak in the DTA thermograms (Figure 2b) [15,16]. Weight loss was observed above 600 °C and can be attributed to complete removal of material during calcination. This weight loss corresponds to an exothermic peak between 600–800 °C observed by DTA for the Co/NS-Al-A and Co/NS-Al-B catalysts (Figure 2b). Both the TGA and DTA analysis of all the catalysts exhibited some minor decomposition up to 900 °C, which clearly implies that all of the catalysts in this study have good thermal stabilities.

**Figure 3:**
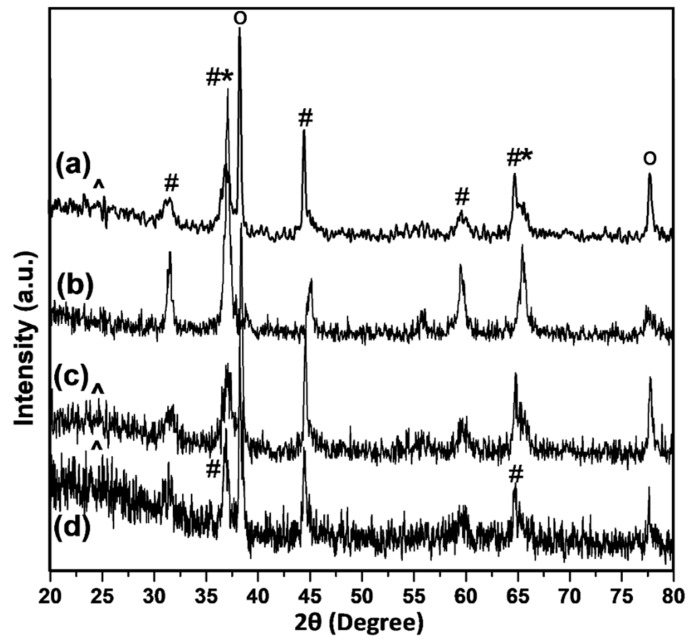
X-ray powder diffraction (XRD) patterns for the calcined of catalysts: (**a**) Co/NS-Al-B, (**b**) Co/Al_2_O_3_, (**c**) Co/NS-Al-A and (**d**) Co/NS catalysts. (*) CoAl_2_O_4_; (#) Co_3_O_4_; (^) SiO_2_; and (o) Co.

The XRD diffractograms of the calcined Co/NS, Co/Al_2_O_3_, Co/NS-Al-A and Co/NS-Al-B catalysts are displayed in Figure 3. The crystalline phase of the Co/NS-Al-A and of the Co/NS-Al-B catalysts were identified by comparisons with the Co/NS and Co/Al_2_O_3_ catalysts. The XRD patterns of the calcined Co/Al_2_O_3_, Co/NS-Al-A and Co/NS-Al-B catalysts show several diffraction peaks. The 2θ diffraction peaks of the Co/Al_2_O_3_, Co/NS-Al-A and Co/NS-Al-B catalysts are approximately 31.7°, 36.9°, 45.0°, 60.1°, 66.0° and 77.8°, and can be attributed to the characteristic diffraction peaks of the Co_3_O_4_ and CoAl_2_O_3_ species [17]. All calcined catalysts show the characteristic reflection peak at around 2θ = 36.9° that corresponds to Co_3_O_4_. Furthermore, all catalysts also showed peaks at 2θ = 38.3° and 77.3°, which were attributed to CoO with cubical structure [18]. The diffraction peaks of Co_3_O_4_ are very close to that of CoAl_2_O_4_ (2θ = 37.0°, 65.0°). The peaks assigned to either Co_3_O_4_ or CoAl_2_O_4_ were observed in the Co/Al_2_O_3_, Co/NS-Al-A and Co/NS-Al-B catalysts. Similar findings were reported by Saraswat et al. [18] and Lee et al. [19]. A broad diffraction peak assigned to amorphous silica NS was present (2θ ~ 23°) in the diffractograms of catalysts containing NS (Figure 3) [14]. The size of the Co_3_O_4_ particles was calculated using Equation (4) of the Co_3_O_4_ diffraction peak (311) at 2θ = 36.9°. The average Co_3_O_4_ crystallite size of in the calcined Co/NS, Co/Al_2_O_3_, Co/NS-Al-A and Co/NS-Al-B catalysts were found to be 9.8, 12.2, 10.6 and 8.6 nm, respectively, and are summarized in Table 1. The Co_3_O_4_ crystalline size of the Co/NS-Al-B catalyst was the smallest for the NS modified with Al_2_O_3_ support. We postulate that this is due to the stronger interaction between the cobalt oxide phase, Al_2_O_3_ modified NS support and the high surface area, resulting in higher catalytic activity [6].

**Figure 4:**
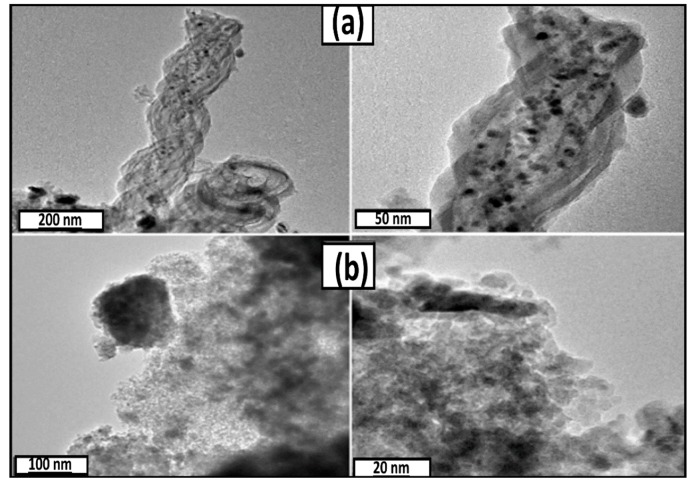

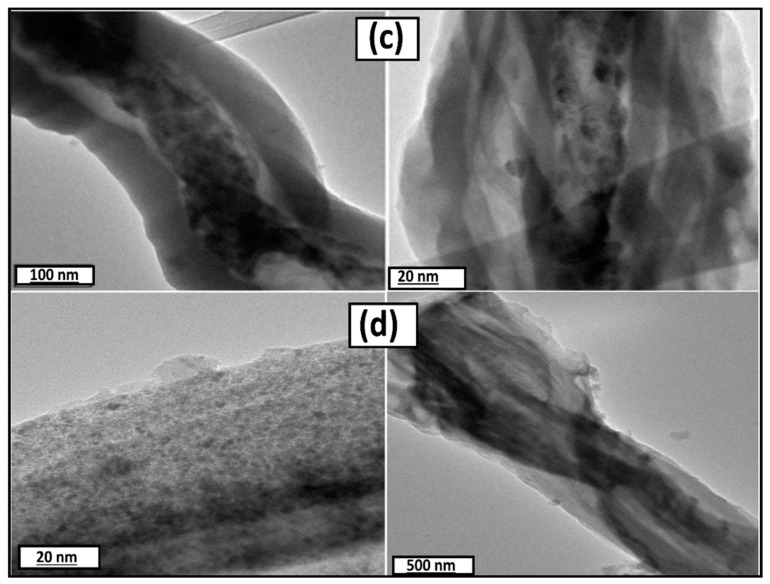
Transmission electron micrographs of the calcined (**a**) Co/NS, (**b**) Co/Al_2_O_3_, (**c**) Co/NS-Al-A and (**d**) Co/NS-Al-B catalysts.

TEM was employed to determine the particle sizes and morphologies of calcined catalysts (Figure 4). The dark spots in the micrographs are Co particles dispersed on the NS surface (Figure 4). The nano-helical structure of the NS support is clearly observed in the micrographs of the Co/NS, Co/NS-Al-A and Co/NS-Al-B catalysts. The Co nanoparticles are well dispersed on the NS for the Co/NS (Figure 4a), Co/NS-AL-A (Figure 4c) and Co/NS-AL-B (Figure 4d) catalysts, whereas for Co/Al_2_O_3_ (Figure 4b) the Co particles were not well dispersed with some agglomerates. Moreover, it was found that the average Co particle size in the Co/NS, Co/Al_2_O_3_, Co/NS-Al-A and Co/NS-Al-B catalysts were 6.6 nm, 10.8 nm, 8.2 nm and 5.3 nm, respectively (Table 1). Thus, the addition of NS as support during the preparation process decreased the average Co particle size in the Co/NS-Al-B catalyst, consistent with the results obtained by XRD.

**Figure 5:**
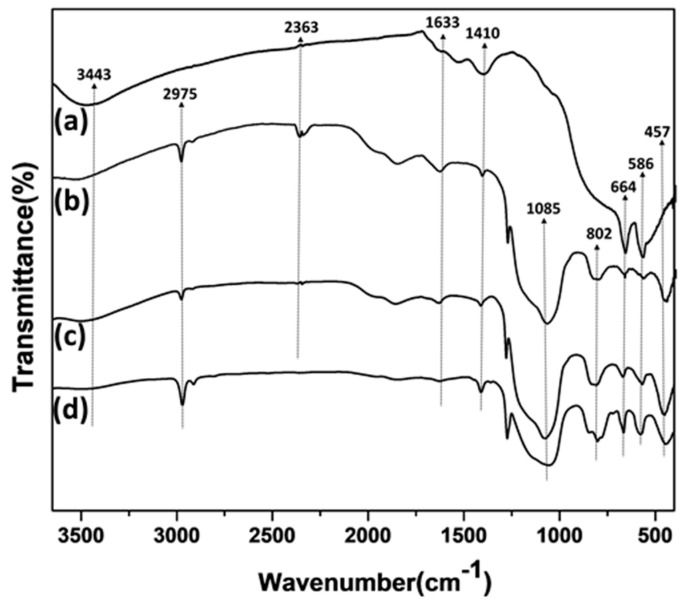
FTIR spectra of the (**a**) Co/Al_2_O_3_, (**b**) Co/NS-Al-A, (**c**) Co/NS-Al-B and (**d**) Co/NS catalysts.

The chemistry of the catalysts was examined by FTIR spectroscopy (Figure 5). Although the FTIR spectra of the Co/NS, Co/NS-Al-A and Co/NS-Al-B catalysts were similar, some differences in intensity were observed with the addition of Al_2_O_3_. The FTIR spectra of the Co/NS, Co/NS-Al-A and Co/NS-Al-B catalysts all showed characteristic Si–O–Si antisymmetric stretching and Si–O symmetric stretching vibrations at approximately 1085 cm^−1^ and 802 cm^−1^, respectively [1,4]. An additional band at 457 cm^−1^ is assigned to Si–O–Si or O–Si–O bending vibrations. The two absorption bands at approximately 586 cm^−1^ and 664 cm^−1^ in all catalysts have been assigned to Co–O [14]. Moreover, all catalysts exhibit a broad band centered at 3443 cm^−1^ associated with O–H stretching, and a relatively weak band at 1633 cm^−1^ of hydrogen bonded surface silanol groups and physically adsorbed water [16,20,21]. The spectra of the Co/Al_2_O_3_, Co/NS-Al-A and Co/NS-Al-B catalysts showed two bands at 567 and 663 cm^−1^, which have been attributed to CoAl_2_O_4_ [22].

The H_2_-TPR was used to investigate the reduction behavior of the as-prepared catalysts. It is well known that the reduction of Co_3_O_4_ species with H_2_ follows a three stage process:

3CoO(OH) + 0.5 H_2_→ Co_3_O_4_ + 2H_2_O (7)

Co_3_O_4_ + H_2_→3CoO+ H_2_O (8)

3CoO + 3H_2_→3Co + 3H_2_O (9)

**Figure 6:**
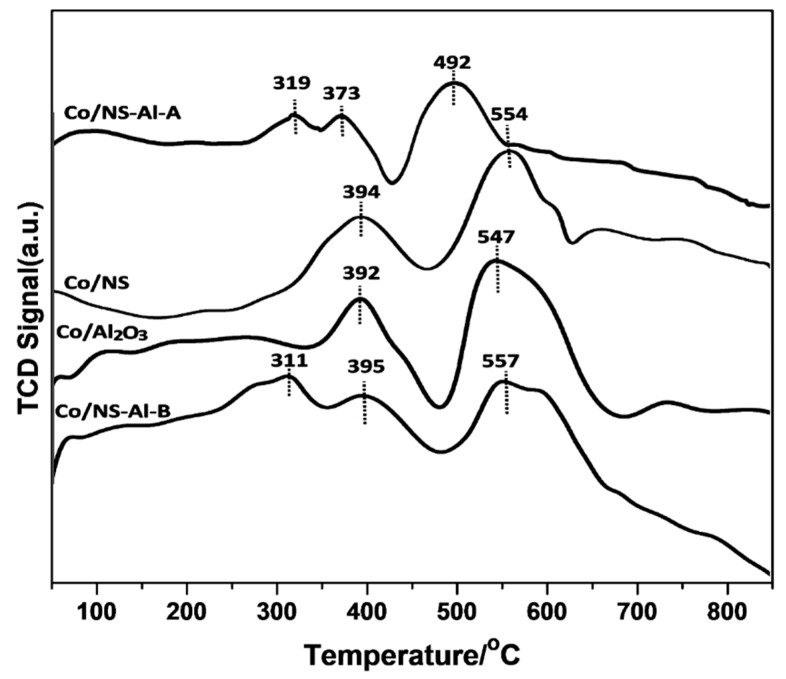
H_2_-TPR profiles for the Co/NS-Al_2_O_3_, Co/NS, Co/Al-A and Co/Al-B catalysts.

The reducibility of metal oxides is known to play a major role in determining FTS activity and product selectivity [1]. Displayed in Figure 6 are the H_2_-TPR profiles of the calcined Co/NS, Co/Al_2_O_3_, Co/NS-Al-A and Co/NS-Al-B catalysts. It can be seen from the H_2_-TPR profiles of the Co/NS and Co/Al_2_O_3_ catalysts that two main peaks occur at 394 and 553 °C, and at 393 and 547 °C, respectively. The low temperature reduction peaks (394 and 393 °C) are attributed to the reduction of Co^3+^ to Co^2+^, whereas the peak areas at the high reduction temperature (553 and 547 °C) are assigned to the reduction of Co^2+^ to Co^0^ metal. In the TPR profiles, peaks were observed at 319 °C, 373 °C and 492 °C for the Co/NS-Al-A catalyst, and for the Co/NS-Al-B catalysts, they were at 311 °C, 395 °C and 557 °C. The low temperature peak in both catalysts is assigned to either the reductive decomposition of residual nitrate species or the reduction of cobalt-oxyhydroxide (CoOOH) species [23]. The two higher temperature peaks at 373 °C and 492 °C for the Co/NS-Al-A catalyst and 395 °C and 557 °C for the Co/NS-Al-B catalyst are due to the reduction of Co_3_O_4_ to CoO and CoO to Co^0^, respectively. The results obtained herein are in general agreement with data previously reported [1,14]. These results demonstrate that the preparation method for the surface coating of the NS with Al_2_O_3_ impacted their reduction temperatures. That is to say, the Co/NS-Al-A catalyst had lower reduction temperatures than the Co/NS-Al-B catalyst, which could be due to weaker interfacial interactions between the Co and silica of the NS. Nevertheless, the TPR profiles for all calcined catalysts clearly show that the activation temperature occurs between 550 and 700 °C and is sufficient to obtain metallic Co.

**Figure 7:**
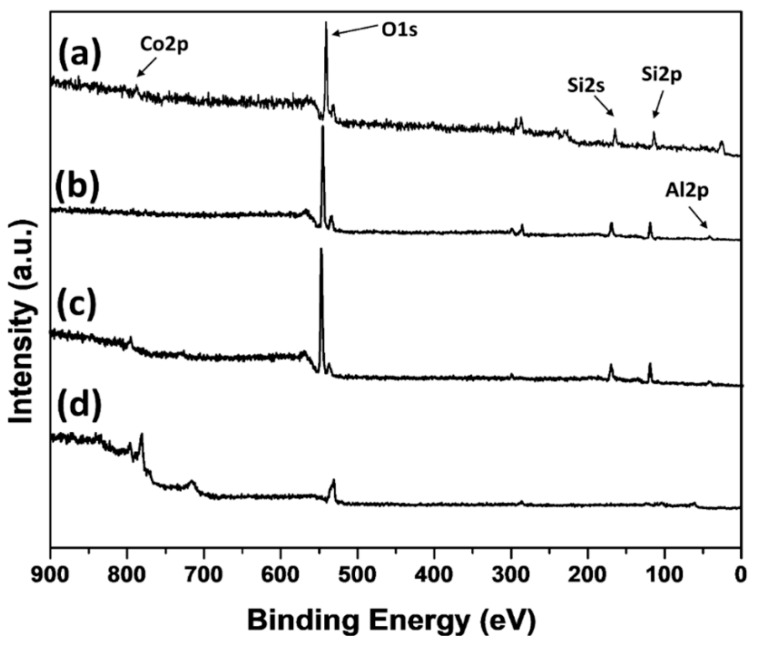
Survey XPS spectra of the calcined (**a**) Co/NS, (**b**) Co/NS-Al-A, (**c**) Co/NS-Al-B and (**d**) Co/Al_2_O_3_ catalysts.

**Figure 8:**
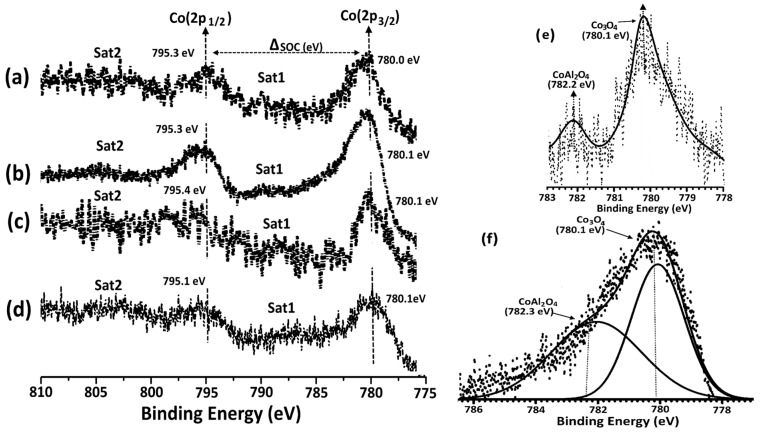
High resolution XPS spectra of the Co2p of the calcined Co/NS (**a**), Co/Al_2_O_3_ (**b**), Co/NS-Al-A (**c**) and Co/NS-Al-B (**d**) catalysts, and expanded region showing CoAl_2_O_4_ for the calcined (**e**) Co/NS-Al-A and (**f**) Co/NS-Al-B catalysts.

XPS measurements were carried out to investigate the surface chemical nature of the catalysts. The XPS spectra of the Co/NS, Co/NS-Al_2_O_3_, Co/Al-A and Co/Al-B catalysts are displayed in Figure 7. The XPS survey scan for all catalysts presents photoelectron lines corresponding to C 1s, O1s, Co 2p, Si 2p and Si 2s plus Al2p for the Co/Al_2_O_3_, Co/Al-A and Co/Al-B catalysts. The Co 2p spectra of all the calcined catalysts are shown in Figure 8. All spectra exhibit two main Co2p_3/2_ and Co2p_1/2_ peaks and two satellites peaks. Characteristic Co 2p_3/2_ and Co2p_1/2_ peaks of the calcined Co/NS, Co/Al_2_O_3_, Co/NS-Al-A and Co/NS-Al-B catalysts were observed at binding energies of 780.0 eV and 795.3 eV, 780.1 eV and 795.3eV, 780.1 eV and 795.4eV and 780.1 eV and 795.1 eV, respectively. Moreover, the spin-orbit coupling (Δ_SOC_) between the Co 2p_3/2_ and Co 2p_1/2_ in Co/NS, Co/Al_2_O_3_, Co/NS-Al-A and Co/NS-Al-B catalysts were 15.3 and 15.2 eV, 15.3 eV and 15.0 eV, respectively. Therefore, the weak satellite peaks observed for all the catalysts indicate that at the oxidation state of the catalysts are Co_3_O_4_ phase, and this is in accordance with the literature [14,22]. The peak in the Co/NS-Al-A and Co/NS-Al-B catalysts at 782.2eV and 782.3 eV is tentatively assigned to the component of CoAl_2_O_4_ [22]. The XPS results suggest that the surface structure of Co/NS is significantly influenced by the modification of NS with Al_2_O_3_ support, and the result of these values are in good agreement with the XRD analysis. Overall, by using XRD, XPS and FTIR data, nanoparticles of Co_3_O_4_ are found to be the dominant phase of the prepared catalysts, where the presence of CoAl_2_O_4_ was observed in both the Co/NS-Al-A and Co/NS-Al-B catalysts.

### 3.2. Catalytic Activity Testing

**Figure 9:**
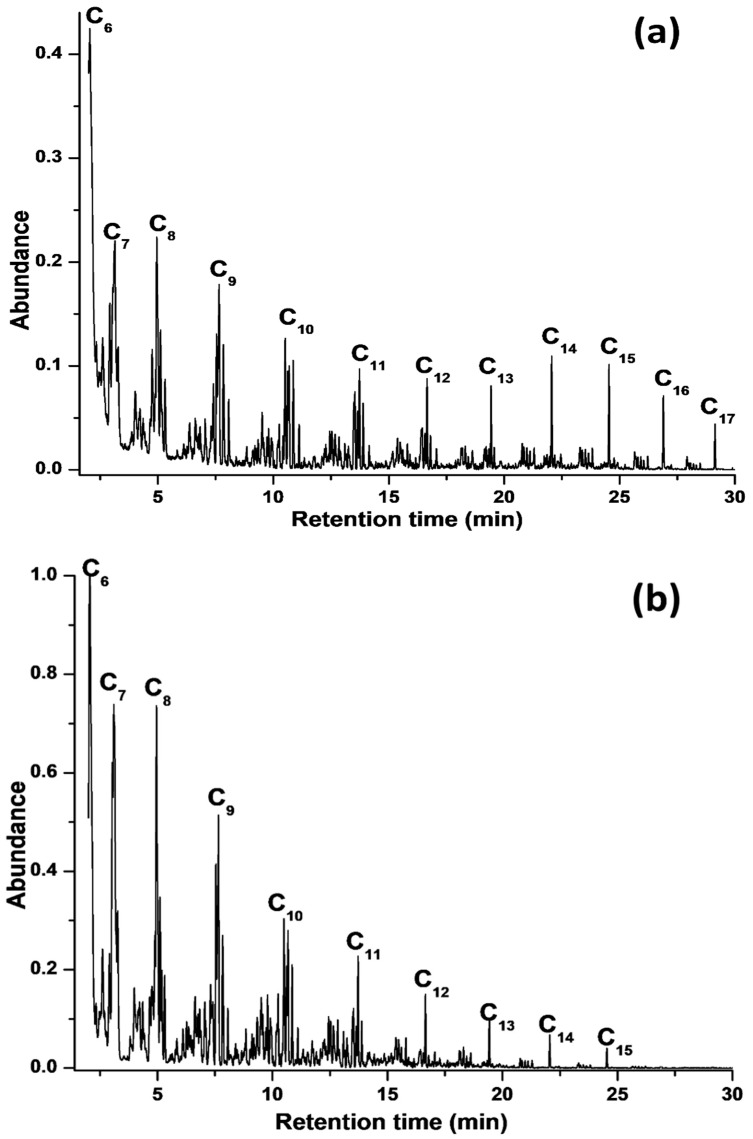
GC-MS chromatograms of FTS liquid products using the catalyst (**a**) Co/NS-Al-B and (**b**) Co/NS-Al-A.

FTS testing (condensable liquid products) of the catalysts was analyzed by GC-MS (Figure 9 and Supplementary Tables S1–S4), while the non-condensable gases (CO, CO_2_, H_2_, N_2_, CH_4_ and C_2_-C_4_) were analyzed by GC. The FTS activities–carbon selectivity to different product ranges and the paraffin to olefin ratios over calcined catalysts have been determined (Table 2). Based on GC and GC-MS analysis, the FTS activity of the Co/NS, Co/Al_2_O_3_, Co/NS-Al-A and Co/NS-Al-B catalysts were 65.5%, 46.8%, 62.8% and 82.4%, respectively. The Co/Al_2_O_3_ catalyst exhibited the lowest activity of 46.8% CO conversion, which was possibly attributable to having the lowest S_BET_ (108 m^2^/g), lowest Co dispersion (10.5%) and largest Co particle size (12.2 nm), relative to the other catalysts (Table 1). It is well known that decreasing the active phase particle size can improve the FTS catalytic performance, control their selectivity and stability [4]. It was noted that no difference in activity was observed between the Co/NS and when it was coated with Al_2_O_3_ to form the Co/NS-Al-A catalyst. However, there was a distinct change in hydrocarbon product distribution Σ < C_5_ (light weight hydrocarbons), CO_2_ and CH_4_ undesirable selectivity going from the Co/NS to the Co/NS-AL-A catalysts (Table 2). However, the Co/NS-Al-B catalyst (NS coated with Al_2_O_3_ and then decorated with Co) showed significantly higher FTS activities (Σ < C_5_, CO_2_ and CH_4_ selectivities) compared to the Co/NS-Al-A catalyst (Table 2). For the Co/NS-Al-B catalyst, the total CO_2_ (0.6%) and Σ < C_5_ selectivity (5.4%) was the lowest of the catalysts in this study. This suggests that the improved Co dispersion (16%), largest S_BET_ (260 m^2^/g) and smaller Co particles (5.7 nm) all contributed to its overall improved performance [3]. Several research groups have studied the effect of the addition of Al_2_O_3_ on the catalytic performance of the Co/SiO_2_ catalysts. For instance, Rathousky et al. [24] examined the influence of Al_2_O_3_ on catalytic performance of the Co/SiO_2_ catalyst, and found that the catalytic behavior of 10% Co/SiO_2_-Al_2_O_3_ was more similar to Co/SiO_2_ than to Co/Al_2_O_3_.

**Figure 10:**
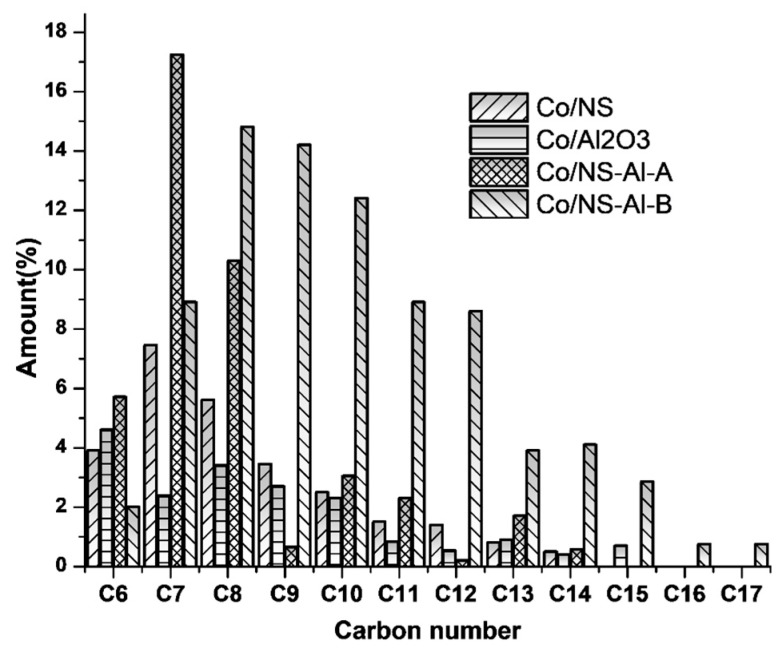
Production distribution of FT hydrocarbons (C_6_–C_14_) of the calcined Co/NS, Co/Al_2_O_3_, Co/NS-Al-A and Co/NS-Al-B catalysts.

The condensable liquid fuel products were characterized by GC-MS (Figure 9), where the Co/NS and Co/Al_2_O_3_ catalysts have a hydrocarbon product distribution in the carbon number range of C_6_–C_14_ (Figure 10), while the Co/NS-Al-A and Co/NS-Al-B catalysts had hydrocarbons ranges of C_6_–C_15_ (naphtha fraction) and C_6_–C_17_, respectively (Figure 10)_._ The FTS hydrocarbons products obtained from the Co/NS-Al-A catalyst were found to be qualitatively and quantitatively different from those produced by the Co/NS-Al-B catalyst (Table 2). The main hydrocarbon products in the C_6_–C_15_ range for the Co/NS-Al-A catalyst were olefins (23.7%), paraffins (8.9%), naphthenes (cycloalkanes) (16.9%) and aromatics (10.2%), giving it a total hydrocarbon selectivity of 58.5%. In addition, there were no oxygenated products detected for the Co/NS-Al-A catalyst. In contrast, the Co/NS-Al-B catalyst produced paraffins (16.2%), olefins (55.1%), naphthenes (12.6%) and some oxygenated products (2.4%), giving it a total hydrocarbons selectivity of 86.3%. In general, the amount of produced olefins in all catalysts was higher than paraffins. Oxygenated products were observed for the Co/NS, Co/Al_2_O_3_ and Co/NS-Al-B catalysts.

Surprisingly, the Co/NS catalyst coated with Al_2_O_3_ (Co/NS-Al-A) yielded C_6_–C_15_ aromatic compounds (10.2%), mainly comprised of mono- and di-nuclear aromatics that include o-xylenes, alkyl-benzenes, naphthalene and alkyl-naphthalene isomers. It is believed that coating the Co/NS catalyst with Al_2_O_3_ (Co/NS-Al-A) may convert the primary products, including olefins and oxygenates, to aromatics via secondary reactions. The olefin/paraffin (O/P) ratio was the least for the Co/NS catalyst (1.44) and increased with the presence of Al_2_O_3_ (Table 2). The Co/NS-Al-B had the highest O/P of 3.40. This increase in olefin content is likely attributable to a reduced hydrogenation rate [14]. The data reported above clearly shows that the addition of Al_2_O_3_ to the NS support, in both the Co/NS-Al-A and Co/NS-Al-B catalysts, has an impact on the product selectivity and distribution of C_6_–C_14_ aromatics. This could be attributable to changes in the surface coverage and morphology rather than a change in the intrinsic activity of the active Co sites [25]. The addition of a small amount of Al_2_O_3_ to the silica-supported Co catalyst significantly improved the dispersion of Co and led to an increase in the Fischer−Tropsch synthesis (FTS) activity, which has been observed by others [5,6]. Furthermore, the Co/NS-Al-B catalyst was superior to the traditional Co-Al_2_O_3_, Co/SiO_2_ and Co/SiO_2_-Al_2_O_3_ catalysts in terms of hydrocarbon distribution Σ > C_6_ and resistant to coke formation.

**Figure 11:**
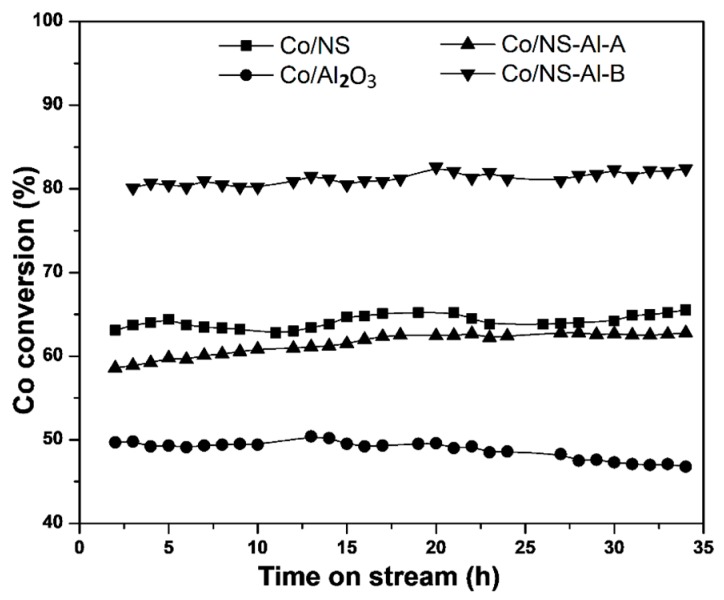
CO conversion as a function of the time on stream for the calcined Co/NS, Co/Al_2_O_3_, Co/NS-Al-A and Co/NS-Al-B catalysts.

The variation of the CO conversion with reaction time can be used as an indicator of catalyst stability (Figure 11). The CO conversion in all catalysts was relatively stable over a 34 h period, with the exception of the Co/Al_2_O_3_ catalyst, which showed a slight decrease from 50% to 47% CO conversion. These results demonstrate that FTS catalysts were stable.

## 4. Conclusions

Catalytic performance of two kinds of Co/NS-Al_2_O_3_ (Co/NS-Al-A and Co/NS-Al-B) catalysts were evaluated in a quartz fix-bed micro-reactor and compared with Co/NS and Co/Al_2_O_3_ catalysts. It was found that the combination of Al_2_O_3_ onto the Co/NS catalyst had a remarkable change in the hydrocarbon product selectivity in both the Co/NS-Al-A and Co/NS-Al-B catalysts. The highest CO conversion was achieved using the Co/NS-Al-B catalyst, which also had the highest surface area and Co dispersion. The product distribution towards the formation of aromatic compounds was formed by adding Al_2_O_3_ to the NS support; also, light hydrocarbon products, CO_2_ and CH_4_, selectivity increased. However, the addition of Al_2_O_3_ in the Co/NS-Al-A catalyst does not appear to have any significant influence on the physical properties of catalysts, such as dispersion, reducibility, average particle size and surface area. From these results, we conclude that the effect of coating Al_2_O_3_ onto the NS catalyst and then impregnating with cobalt (Co/NS-Al-B catalyst) can be attributed to structural rearrangement of the Co surface, not to a change in the intrinsic activity. In general, our findings have implications for designing modified catalysts that can decrease the oxygenated compound and increase aromatic content, and this represents a fertile area for further research.

## Figures and Tables

**Table 1:** Surface area (S_BET_) of calcined catalysts.

Catalysts	Al (wt.%)	Co (wt.%)	S_BET_ (m^2^ g^−1^)	Size of Co_3_O_4_ Particles (nm)		d_XRD_ (Co^0^) (nm)	Co Dispersion (%)
				*d*_XRD_	*d*_TEM_		
NS	-	-	314	-	-	-	-
Al_2_O_3_	-	-	227	-	-	-	-
Co/NS	-	15	193	9.8	6.6	7.3	13.1
Co/Al_2_O_3_	85	15	108	12.2	10.8	9.1	10.5
Co/NS-Al_-_A Co/NS-Al_-_B	8 8	15 15	199 260	10.6 8.1	8.2 5.7	7.8 6.0	12.3 16.0

**Table 2:** Catalytic performance and major components of synthesized liquid FTS fuel over the calcined Co/NS, Co/Al_2_O_3_, Co/NS-Al-A and Co/NS-Al-B catalysts at 230 °C, H_2_/CO = 2 and at atmospheric pressure.

Catalyst	Co/NS	Co/Al_2_O_3_	Co/NS-Al-A	Co/NS-Al-B
CO Conversion (%)	65.5	46.8	62.8	82.4
H_2_ Conversion (%)	61.2	39.7	56.2	73.3
Products Selectivity (%)				
CO_2_ select. (%)	5.3	17.4	8.7	0.6
CH_4_ select. (%)	6.7	20.4	10.6	7.7
Σ < C_5_	17.1	29.6	21.0	5.4
Product distribution (Mol. %)				
Σ > C_6_	70.9	32.6	58.5	86.3
Paraffins	18.4	5.8	8.9	16.2
Olefins	26.6	13.6	23.7	55.1
Naphthenes	17.3	8.6	16.9	12.6
Oxygenates	8.6	4.6	-	2.4
Aromatics	-	-	10.2	-
Olefins /Paraffins (O/P)	1.44	2.34	2.66	3.40

## Supplementary Materials

The following are available online at https://www.mdpi.com/1996-1944/12/11/1810/s1. Table S1: The FT products identified by GC-MS for unmodified Co/NS catalyst reduced by H_2_ at a temperature of 230 °C and H_2_/CO = 2; Table S2: The FT products identified by GC-MS for unmodified Co/Al_2_O_3_ catalyst reduced by H_2_ at a temperature of 230 °C and H_2_/CO = 2; Table S3: The FT products identified by GC-MS for unmodified Co/NS-Al-A catalyst reduced by H_2_ at a temperature of 230 °C and H_2_/CO = 2; Table S4: The FT products identified by GC-MS for modified Co/NS-Al-B catalyst reduced by H_2_ at a temperature of 230 °C and H_2_/CO = 2; Figure S1: TEM micrographs (**a** and **b**) of prepared silica nanosprings (NS) as support (without catalyst).

## References

[1] Alayat A., Mcllroy D., McDonald A.G. (2018). Effect of synthesis and activation methods on the catalytic properties of silica nanospring (NS)-supported iron catalyst for Fischer-Tropsch synthesis. Fuel Process. Technol..

[2] Kengne B.-A.F., Alayat A.M., Luo G., McDonald A.G., Brown J., Smotherman H., McIlroy D.N. (2015). Preparation, surface characterization and performance of a Fischer-Tropsch catalyst of cobalt supported on silica nanosprings. Appl. Surf. Sci..

[3] Bao A., Liew K., Li J. (2009). Fischer-Tropsch synthesis on CaO-promoted Co/Al_2_O_3_ catalysts. J. Mol. Catal. A Chem..

[4] Alayat A., Echeverria E., Mcllroy D.N., McDonald A.G. (2018). Enhancement of the catalytic performance of silica nanosprings (NS)-supported iron catalyst with copper, molybdenum, cobalt and ruthenium promoters for Fischer-Tropsch synthesis. Fuel Process. Technol..

[5] Savost’yanov A.P., Yakovenko R.E., Sulima S.I., Bakun V.G., Narochnyi G.B., Chernyshev V.M., Mitchenko S.A. (2017). The impact of Al_2_O_3_ promoter on an efficiency of C^5+^ hydrocarbons formation over Co/SiO_2_ catalysts via Fischer-Tropsch synthesis. Catal. Today.

[6] Zhang Y., Nagamori S., Hinchiranan S., Vitidsant T., Tsubaki N. (2006). Promotional effects of Al_2_O_3_ addition to Co/SiO_2_ catalysts for Fischer-Tropsch synthesis. Energy Fuels.

[7] Prasongthum N., Reubroycharoen P. (2015). Preparation of Co/SiO_2_-Al_2_O_3_ Fiber Catalyst by Electrospinning for Fischer-Tropsch Synthesis. Key Eng. Mater..

[8] Heidarinasab A., Soltanieh M., Ardjmand M., Ahmadpanahi H., Bahmani M. (2016). Comparison of Mo/MgO and Mo/γ-Al_2_O_3_ catalysts: Impact of support on the structure and dibenzothiophene hydrodesulfurization reaction pathways. Int. J. Environ. Sci. Technol..

[9] Luo G., Fouetio Kengne B.A., McIlroy D.N., McDonald A.G. (2014). A novel nano fischer-tropsch catalyst for the production of hydrocarbons. Environ. Prog. Sustain. Energy.

[10] Ji L., Lin J., Tan K., Zeng H. (2000). Synthesis of high-surface-area alumina using aluminum tri-sec-butoxide−2, 4-pentanedione−2-propanol− nitric acid precursors. Chem. Mater..

[11] Ahmadipour M., Hatami M., Rao K.V. (2012). Preparation and characterization of nano-sized (Mg_(x)_Fe_(1–x)_O/SiO_2_)(x = 0.1) core-shell nanoparticles by chemical precipitation method. Adv. Nanopart..

[12] Hao Q.-Q., Zhao Y.-H., Yang H.-H., Liu Z.-T., Liu Z.-W. (2012). Alumina grafted to SBA-15 in supercritical CO_2_ as a support of cobalt for Fischer-Tropsch synthesis. Energy Fuels.

[13] Zhao Y.-H., Song Y.-H., Hao Q.-Q., Wang Y.-J., Wang W., Liu Z.-T., Zhang D., Liu Z.-W., Zhang Q.-J., Lu J. (2015). Cobalt-supported carbon and alumina co-pillared montmorillonite for Fischer-Tropsch synthesis. Fuel Process. Technol..

[14] Alayat A.M., Echeverria E., Mcllroy D.N., McDonald A.G. (2018). Characterization and catalytic behavior of EDTA modified silica nanosprings (NS)-supported cobalt catalyst for Fischer-Tropsch CO-hydrogenation. J. Fuel Chem. Technol..

[15] Trotte N.S., Aben-Athar M.T., Carvalho N.M. (2016). Yerba Mate Tea Extract: A Green Approach for the Synthesis of Silica Supported Iron Nanoparticles for Dye Degradation. J. Braz. Chem. Soc..

[16] Rafiee H.R., Feyzi M., Jafari F., Safari B. (2013). Preparation and Characterization of Promoted Fe-V/SiO_2_ Nanocatalysts for Oxidation of Alcohols. J. Chem..

[17] Nuernberg G.B., Fajardo H.V., Mezalira D.Z., Casarin T.J., Probst L.F., Carreño N.L. (2008). Preparation and evaluation of Co/Al_2_O_3_ catalysts in the production of hydrogen from thermo-catalytic decomposition of methane: Influence of operating conditions on catalyst performance. Fuel.

[18] Saraswat S.K., Pant K. (2015). Progressive Loading Effect of Co Over SiO_2_/Al_2_O_3_ Catalyst for Cox Free Hydrogen and Carbon Nanotubes Production Via Catalytic Decomposition of Methane. Progressive.

[19] Lee G.-Y., Ryu K.-H., Kim H.-G., Kim Y.-Y. (2009). The Preparation of Blue CoAl_2_O_4_ Powders by the Malonate Method: The Effect of the Amount of Malonic Acid Used, the Formation Pathway of CoAl_2_O_4_ Crystallites and the Characteristics of the Prepared Powders. Bull. Korean Chem. Soc..

[20] Moghanian H., Mobinikhaledi A., Blackman A., Sarough-Farahani E. (2014). Sulfanilic acid-functionalized silica-coated magnetite nanoparticles as an efficient, reusable and magnetically separable catalyst for the solvent-free synthesis of 1-amido-and 1-aminoalkyl-2-naphthols. RSC Adv..

[21] Nabid M.R., Bide Y., Abuali M. (2014). Copper core silver shell nanoparticle–yolk/shell Fe_3_O_4_@ chitosan-derived carbon nanoparticle composite as an efficient catalyst for catalytic epoxidation in water. RSC Adv..

[22] Ji L., Tang S., Chen P., Zeng H., Lin J., Tan K. (2000). Effect of nanostructured supports on catalytic methane decomposition. Pure Appl. Chem..

[23] De Beer M., Kunene A., Nabaho D., Claeys M., Van Steen E. (2014). Technical and economic aspects of promotion of cobalt-based Fischer-Tropsch catalysts by noble metals-a review. J. S. Afr. Inst. Min. Metall..

[24] Zhang J., Chen J., Li Y., Sun Y. (2002). Recent technological developments in cobalt catalysts for Fischer-Tropsch synthesis. J. Nat. Gas Chem..

[25] Rohr F., Lindvåg O., Holmen A., Blekkan E.A. (2000). Fischer-Tropsch synthesis over cobalt catalysts supported on zirconia-modified alumina. Catal. Today.

