# Supplementary material for: Alumina Coated Silica Nanosprings (NS) Support Based Cobalt Catalysts for Liquid Hydrocarbon Fuel Production From Syngas
Source: Materials (Basel). 2019 Jun 4;12(11):1810. doi: 10.3390/ma12111810 (PMC6600709; doi:10.3390/ma12111810)
Supplement: Supplementary file 1 [file materials-12-01810-s001.pdf]

materials

Supplementary

Alumina Coated Silica Nanosprings (NS) Support Based Cobalt Catalysts for Liquid Hydrocarbon Fuel Production From Syngas

Abdulbaset Alayat, Elena Echeverria, Farid Sotoudehniakarani, David N. Mcllroy and Armando G. McDonald *

Table S1. The FT products identified by GC-MS for unmodified Co/NS catalyst reduced by H_2 at a temperature of 230 °C and H_2/CO = 2.

Retention Time (min)	Identified Compounds	Mol(%)	M+ (m/z)
2.16	2-pentene 3-methyl	0.65	84
2.21	3-hexene-(z)	0.72	84
2.29	2-ethyl-1-pentanol	2.09	116
2.38	3-Methylhexane	0.45	100
2.56	cis-1,2 -dimethyl cyclopentane	7.4	98
2.65	Heptane	1.83	100
2.75	3-Heptene	1.21	98
2.77	Isopropylcyclobutane	1.0	98
2.88	Cycloheptane	0.68	98
3.35	Cyclopropane, (1,2-dimethylpropyl)	0.13	112
3.41	3-Methyl-1-heptene	0.78	112
3.54	5-Methyl-1-heptene	1.65	112
3.69	6-Methyl-1-heptene	1.38	130
3.71	1-octanol	1.24	130
3.84	3-Ethyl-4-methyl-1-pentanol	1.24	130
4.12	3,4-Dimethyl-1-hexene	0.38	112
4.20	Cyclopropane, pentyl	5.72	112
4.38	Octane	2.05	114
4.56	1,2-Diethylcyclobutane	0.76	112
4.75	2-Octene	0.48	112
5.54	Cis-3-Nonene	0.52	126
5.75	2,3-Diethyl-3-heptene	1.68	126
5.95	6-Methyl-1-octene	1.34	126
6.16	4-Ethylheptane	1.17	128
6.39	2-Nonene, (E)-	0.27	126
6.57	7-Methyl-1-octene	0.41	126
6.70	1-Nonene	3.37	126
6.93	Nonane	1.62	128
7.13	2-Nonene, (E)-	0.75	126
7.38	Cis-4-Nonene	0.68	126
7.90	2,3,5-Trimethylhexane	0.26	128
8.33	Cyclopropane, 1-methyl-2-pentyl-	0.49	140
8.48	1-Decene	1.09	140
8.74	3,7-Dimethyl-1-octanol	1.09	158
9.01	3-Methylnonane	0.48	142
9.21	cis-4-Decene	0.46	140
9.48	(Z)-2-Decene	0.25	140

9.63	4-Decene	2.62	140
9.73	Cyclopropane, 1-ethyl-2-pentyl-	0.26	140
9.88	Decane	1.63	142
10.09	trans-3-Decene	0.67	140
10.33	2-Methyl-4-nonene	0.66	140
10.37	3,7-Dimethyl-1-octene	0.31	140
11.32	2-Methyl-2-decene	0.17	154
11.45	4-Methyl-1-decene	0.48	154
11.59	2-Undecene, cis-	0.51	154
11.70	2,3,7-Trimethyloctane	0.35	156
11.81	2,5-Dimethylnonane	0.5	156
12.00	3-Methyldecane	0.47	156
12.64	1-Pentyl-2-propylcyclopropane	1.38	154
12.86	Dodecane	1.40	170
12.94	2,6-Dimethyldecane	0.07	168
13.33	cyclopentane, 1-butyl-2-propyl-	0.34	168
13.47	2-pentyl-1-heptene	0.15	168
14.27	4-Undecene, 5-methyl-, (E)-	0.65	168
14.39	4-Undecene, 7-methyl	0.34	168
14.50	4-Ethyldecane	0.47	170
14.74	2-Methyl-1-undecanol	0.49	186
14.80	2-Butyl-1-octanol	0.35	186
14.93	2,3-Dimethyldecane	0.35	170
14.99	5-Ethyl-2,2,3-trimethylheptane	0.20	170
15.55	Cyclopropane, 1-hexyl-2-propyl-	0.89	168
15.77	Tridecane	12.9	184
15.97	4-Dodecene, (E)-	0.48	168
16.20	2-Butyl-1-octanol	0.29	186
16.26	2-Dodecene, (E)-	0.03	168
17.23	1-Dodecanol, 2-methyl-	0.21	200
17.41	2,3-Dimethylundecane	0.25	184
17.72	2,5,9-Trimethyldecane	0.20	184
17.88	6-Tridecene	0.21	182
18.33	Cyclotridecane	0.38	182
18.52	Tridecane	0.73	184
18.61	2,6,8-Trimethyldecane	0.06	184
18.71	2-Methyldodecan-1-ol	0.27	200
18.86	2,5-Dimethyldodecane	0.03	198
19.95	2,6,7-Trimethyldecane	0.26	198
21.13	3-Methyltridecane	0.47	198

Table S2. The FT products identified by GC-MS for unmodified Co/Al_2O_3 catalyst reduced by H_2 at a temperature of 230 °C and H_2/CO = 2.

Retention Time (min)	Identified Compounds	Mol(%)	M+ (m/z)
2.15	3-Methyl-2-pentene	4.02	84
3.20	3-Methyl-3-hexene	2.32	98
3.35	Cyclohexane	0.52	84
4.12	1,8-Nonadien-3-ol	0.45	140
5.11	3-Methyl-1-heptanol	2.30	130
5.36	3-Methyl-4-methylenehexane	0.48	112
5.48	Cyclobutane, 1,2-diethyl-, trans	0.23	112
6.85	2,3,5-Trimethylhexane	0.45	128
6.94	2-methyl-3-methylene-heptane	0.24	128
7.53	4-Methyl-3-heptene	0.23	126
7.76	4-Nonene	0.56	126
7.87	2,4,6-Trimethylheptane	1.80	142
8.07	Cyclopropane, 1-methyl-2-pentyl	0.36	126
9.72	3-Ethyl-4-methylpentan-1-ol	0.36	130
10.04	4-Methyl-2-propyl-1-pentanol	0.23	144
11.11	3,7-dimethyl-1-octene	0.15	140
12.01	2,3,4-Trimethylhexane	0.16	128
12.50	(S)-3-Ethyl-4-methylpentanol	0.04	130
12.70	3,4-Dimethyl-1-octene	0.14	140
12.81	4-Methyldecane	0.19	156
13.12	3,4,5-Trimethylheptane	0.15	142
14.00	Undecane	0.43	156
14.51	5-Methyldecane	0.12	156
15.65	2,6-Dimethyldecane	0.02	170
15.74	4,5-Dimethylnonane	0.07	156
16.08	Octane, 3,4,5,6-tetramethyl	0.06	120
16.93	Tridecane	0.36	184
17.34	2,5,9-Trimethyldecane	0.04	184
18.58	4,7-dimethyl undecane	0.03	184
18.72	2-Methyl-6-ethyldecane	0.04	184
18.92	2,2,7-Trimethyldecane	0.05	184
19.20	2, 4-Dimethyl-1-(1-methylethyl)-benzene	0.02	148
19.72	Tetradecane	0.28	198
20.18	2,2,3-Trimethylhexane	0.01	128
21.06	3,5-Dimethylundecane	0.03	184
21.13	3-Methyl-3-ethyldecane	0.02	184
21.59	2,2-Dimethylundecane	0.62	184
24.83	Pentadecane	0.21	212
27.19	2,7,10-Trimethyldodecane	0.09	212
29.43	5,8-Diethyldodecane	0.05	226

Table S3. The FT products identified by GC-MS for unmodified Co/NS-Al-A catalyst reduced by H_2 at a temperature of 230 °C and H_2/CO = 2.

Retention Time (min)	Identified Compounds	Mol(%)	M+ (m/z)
2.52	Benzene	2.99	78
2.96	Cyclopropane, 1,2,3-trimethyl	0.76	84
3.01	3-Ethyl-2-pentene	9.62	98
3.07	Cyclopropane, 1,1-diethyl	0.78	98
3.12	4-Methyl-2-pentene	1.43	84
3.89	2-Methyl-3-ethyl-1-pentene	0.52	112
4.13	1,3,5-Cycloheptatriene	6.86	92
4.57	Heptane, 4-methylene	0.62	112
4.85	5-Methyl-1-heptene	4.03	112
5.08	1,2,4-Trimethylcyclopentane	0.68	112
6.01	3-Ethyl-2-methyl-1-heptene	0.21	140
6.41	Ethylbenzene	0.91	106
6.49	3-Ethylhexane	0.47	114
6.64	o-Xylene	2.51	106
6.97	2-Hexene, (E)-	0.54	84
7.27	1-Pentene, 2,3,3-trimethyl-5-phenyl	1.56	188
7.41	2,3-Dimethyl-1-hexene	0.59	112
7.53	2,3,4-Trimethylhexane	0.21	128
9.37	1-Ethyl-3-methylbenzene	1.73	120
9.66	3,6-Dimethyloctane	0.23	142
9.89	Benzene, (1-methylethyl)-	0.19	120
10.12	4-Nonene, 3-methyl-, (Z)-	0.28	140
10.32	1-Ethyl-2-methylbenzene	0.63	120
10.55	2,4,6-Trimethyloctane	0.74	156
11.59	Indane	0.35	118
11.86	Indene	0.15	116
12.30	Ethylcyclopropane	0.08	86
12.39	3-Ethyl-5-methylheptane	0.20	142
12.70	2,2-Dimethyloctane	0.15	142
12.99	Methyl-2,3-Dihydro-1H-indene	0.11	132
13.13	1-Ethenyl-4-ethylbenzene	0.27	132
13.59	2,5,9-Trimethyldecane	0.55	184
14.71	2,3-Dihydro-5-methyl-1H-indene	0.31	132
15.02	Indene, 1-methyl	0.32	130
15.22	2-Methyl indane	0.21	130
16.02	Naphthalene	0.51	128
16.52	2,5,9-Trimethyldecane	0.41	184
18.19	2,8-Dimethylundecane	0.09	184
18.46	1,2-dihydro-4-methylnaphthalene	0.19	144
18.75	Benzene, pentamethyl	0.11	184
19.13	2-Methylnaphthalene	1.07	142
19.30	2-Methyl-6-ethyldecane	0.58	184
19.61	3-Methylnaphthalene	0.17	142
21.95	2,3,5-Trimethylheptane	0.33	142

Table S4. The FT products identified by GC-MS for modified Co/NS-Al-B catalyst reduced by H_2 at a temperature of 230 °C and H_2/CO = 2.

Retention Time (min)	Identified Compounds	Mol(%)	M+ (m/z)
2.60	2-Methyl-1-pentene	1.91	84
2.91	1-Heptene	1.90	98
3.13	3-Heptene	5.88	98
3.27	Cyclopropane,1,1 diethyl	1.03	98
3.87	Pentane, 2-cyclopropyl	0.39	112
4.01	Cyclopropane, (1,2-dimethylpropyl)	1.44	112
4.22	3-Methyl-1-heptanol	1.01	130
4.35	Heptane, 2-methyl	0.76	114
4.75	1-Octene	1.93	112
4.95	2-Octene	5.61	112
5.11	3-Octene, (3E)-	2.34	112
5.31	4-Octene, (4E)-	1.24	112
6.12	2,3-Dimethyl-2-heptene	0.23	126
6.37	Heptane, 2-methyl-3-methylene	0.98	126
6.61	2,3,4-Trimethylhexane	0.66	128
7.04	4-Nonene	0.88	126
7.31	4-Methyl-3-heptene	0.74	126
7.40	Cis-2-Nonene	1.58	126
7.54	3-Nonene	1.99	126
7.65	Methylpentyl cyclopropane	4.08	126
7.84	Cis-4-Nonene	2.09	126
8.06	2-Nonene	0.95	126
8.38	2,2,6-Trimethylheptane	0.15	156
9.12	4-Nonene, 5-methyl	0.34	140
9.20	Cyclopropane, 1,2-dimethyl-1-pentyl	0.42	140
9.34	2,6-Dimethyl-3-octene	0.42	140
9.52	1-Methyl-2-(3-methylpentyl)cyclopropane	1.40	140
9.72	2,6-Dimethyl-3-octene	0.40	140
9.80	2,5-Dimethyl-2-octene	0.49	140
9.92	4-Nonene, 2-methyl-, (Z)-	0.77	140
10.25	4-Nonene, 5-methyl-	1.07	140
10.51	Cis-4-Decene	2.16	140
10.63	Trans-3-Decene	1.44	140
10.69	Decane	1.28	142
10.86	3-Decene	1.41	140
11.12	2,4-Dimethyl-4-octene	0.55	140
11.79	Nonane, 2-methyl-3-methylene	0.18	154
12.11	4-Decene, 2-methyl-, (E)-	0.19	154
12.27	4-Decene, 7-methyl-, (E)-	0.26	154
12.44	2-Decene, 6-methyl-, (Z)-	0.37	154
12.67	4-Undecene, (E)-	0.71	154
12.85	Decane, 3-methyl-	0.42	154
13.09	Decane, 5-methyl	0.31	154
13.50	4-Undecene, (E)-	2.01	154
13.53	5-Undecene, (E)-	1.0	154
13.66	4-Decene, 2-methyl-, (E)-	0.94	154
13.73	Undecane	1.24	156
13.89	2-Undecene	0.89	154
14.15	4-Decene, 9-methyl-, (E)-	0.27	154

14.43	2,4,6-Trimethyl-1-nonene	0.08	168
15.18	3-Undecene, 7-methyl-, (Z)-	0.42	168
15.30	2-Methyl-2-undecene	0.09	168
15.36	4-Methyl-1-undecene	0.49	168
15.38	1-Decene, 2,4-dimethyl	0.15	168
15.49	4-Methyl-1-undecene	0.51	168
15.61	2-Ethyl-1-decanol	0.52	186
15.69	5-Undecene, 3-methyl-, (Z)-	0.05	186
15.81	4-Methyl-1-undecene	0.29	168
15.92	5-Methyl-1-undecene	0.17	168
15.99	5-methyl-3-undecene	0.08	168
16.16	4-Methyl-5-undecene	0.14	168
16.25	4-Methyl-2-undecene	0.07	168
16.34	2-Methyl-2-undecene	0.05	168
16.41	5-Dodecene, (E)-	0.72	168
16.45	4-Dodecene	0.49	168
16.56	6-Dodecene, (E)-	0.54	168
16.66	Dodecane	1.15	170
16.81	9-Methyl-2-undecene	0.45	168
16.96	8-Methyl-1-undecene	0.05	168
17.06	9-Methyl-1-undecene	0.29	168
17.90	5-methyl-3-undecene	0.07	168
18.14	3,4-Dimethyl-1-decene	0.25	168
18.19	4-Methyl-1-undecene	0.23	168
18.31	2-Ethyl-1-decanol	0.43	186
18.45	3,8-dimethylundecane	0.13	186
18.62	6-Ethyl-2-methyldecane	0.35	184
18.95	4-Methyl-2-dodecen	0.08	182
19.14	6-Tridecene, (E)-	0.39	182
19.61	5-Tridecene, (E)-	1.03	182
19.23	3-Tridecene, (E)-	0.29	182
19.33	6-Tridecene, (Z)-	0.25	182
19.43	Tridecane	1.03	184
19.57	1-Tridecene	0.28	184
20.78	6-Methyltridecane	0.34	198
20.85	5-Methyltridecane	0.31	198
20.89	2,3,5,8-Tetramethyl-decane	0.28	198
21.12	2-methyltridecane	0.25	198
21.30	3-methyltridecane	0.40	198
21.59	6-Methyl Tridecane	0.05	196
21.73	7-Tetradecene	0.13	196
21.79	3-Tetradecene, (Z)-	0.11	196
21.97	7-Ttetradecene (z)-	0.15	196
22.06	Tetradecane	1.45	196
22.19	7-Methyl-6-tridecene	0.19	196
22.31	2,3,5,8-Tetramethyl-decane	0.13	198
22.46	2-Hexyl-1-octanol	0.21	214
23.28	2,7,10-Trimethyldodecane	0.50	212
23.52	4-Methyltetradecane	0.31	212
23.65	2,6,11-Trimethyldodecane	0.25	212
23.82	3-Methyltetradecane	0.23	212
24.54	Pentadecane	1.28	212

26.22	2,7,10-Trimethyldodecane	0.17	212
26.90	Hexadecane	1.02	226
26.67	Pentadecane	0.04	226
29.14	3-Methylhexadecane	0.57	240

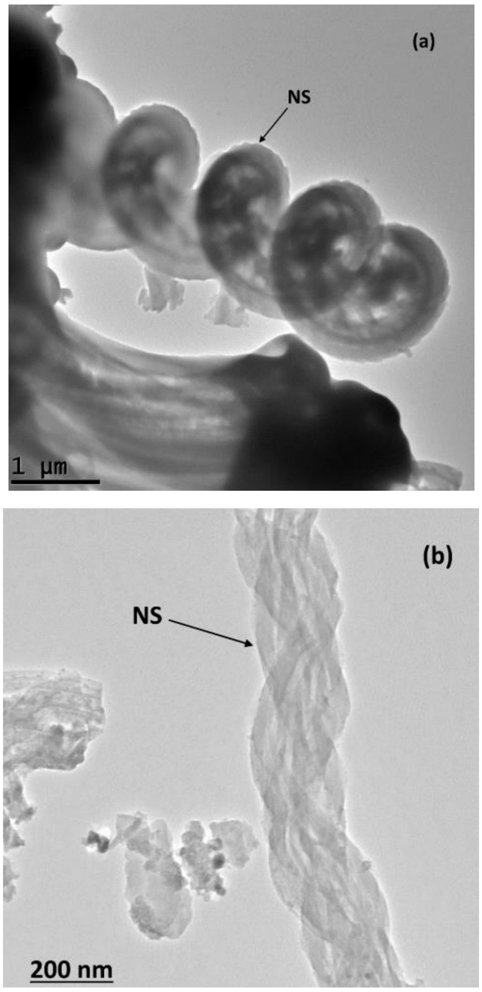

Figure S1. TEM micrographs (**a** and **b**) of prepared silica nanosprings (NS) as support (without catalyst).
